# Self-agglomerated collagen patterns govern cell behaviour

**DOI:** 10.1038/s41598-021-81054-5

**Published:** 2021-01-15

**Authors:** Aysegul Dede Eren, E. Deniz Eren, Twan J. S. Wilting, Jan de Boer, Hanneke Gelderblom, Jasper Foolen

**Affiliations:** 1 Biointerface Science Group, Department of Biomedical Engineering, Institute for Complex Molecular Systems, Eindhoven University of Technology, Eindhoven, The Netherlands; 2 Laboratory of Physical Chemistry, Department of Chemical Engineering and Chemistry, Eindhoven University of Technology, Eindhoven, The Netherlands; 3 Fluids and Flows Group, J.M. Burgers Centre for Fluid Dynamics, Department of Applied Physics, Eindhoven University of Technology, Eindhoven, The Netherlands; 4 Regenerative Engineering & Materials, Department of Biomedical Engineering, Eindhoven University of Technology, Eindhoven, The Netherlands; 5 Institute of Complex Molecular Systems, Eindhoven University of Technology, Eindhoven, The Netherlands

**Keywords:** Cytoskeleton, Biological techniques, Biophysics, Biomaterials, Nanoscale materials, Soft materials

## Abstract

Reciprocity between cells and their surrounding extracellular matrix is one of the main drivers for cellular function and, in turn, matrix maintenance and remodelling. Unravelling how cells respond to their environment is key in understanding mechanisms of health and disease. In all these examples, matrix anisotropy is an important element, since it can alter the cell shape and fate. In this work, the objective is to develop and exploit easy-to-produce platforms that can be used to study the cellular response to natural proteins assembled into diverse topographical cues. We demonstrate a robust and simple approach to form collagen substrates with different topographies by evaporating droplets of a collagen solution. Upon evaporation of the collagen solution, a stain of collagen is left behind, composed of three regions with a distinct pattern: an isotropic region, a concentric ring pattern, and a radially oriented region. The formation and size of these regions can be controlled by the evaporation rate of the droplet and initial collagen concentration. The patterns form topographical cues inducing a pattern-specific cell (tenocyte) morphology, density, and proliferation. Rapid and cost-effective production of different self-agglomerated collagen topographies and their interfaces enables further study of the cell shape-phenotype relationship in vitro. Substrate topography and in analogy tissue architecture remains a cue that can and will be used to steer and understand cell function in vitro, which in turn can be applied in vivo, e.g. in optimizing tissue engineering applications.

## Introduction

Cell morphology is affected by its physical environment, which was shown to strongly modulate cell fate. For instance, healthy tendon tissue is composed of highly ordered anisotropic collagen fibers and tenocytes adopt a spindle-like cell morphology and ultimately, functionally contribute to tissue homeostasis^1^. In tendinopathy, however, tissue organization is disrupted and, hence, the collagen network becomes more isotropic. Concordantly, tenocyte morphology transforms into a stellate-shape, and they change their proliferation speed and produce matrix proteins that compromise tendon function^2^. Various in vitro platforms have been developed to study the relation between matrix structure or substrate topography and cell morphology and the resulting downstream responses. Production of such in vitro platforms often requires the use of advanced tools such as micro-contact printing^3^, electrospinning^4^, or dip-pen nanolithography^5^. Whilst these tools allow the generation of a wide variety of surface patterns that can induce an elongated cell morphology, they are also time-consuming and expensive. Therefore, there is a need for a simple, fast, and cheap method to produce topographies that can induce specific morphologies of tenocytes (spindle-shaped versus stellate-shaped) to study the cell shape-fate relation.

Evaporation of a liquid solution droplet is a simple, yet powerful technique to assemble non-volatile solutes into highly-ordered structures^6^. When a liquid droplet containing colloidal particles or polymers evaporates, it leaves behind a distinct and reproducible pattern, such as the well-known coffee stain^7^. The key behind this remarkably robust pattern formation is an evaporation-driven capillary flow that transports particles towards the contact-line of the droplet^7,8^. The local increase in solute concentration near the contact line subsequently leads to jamming^9^, crystallisation^10^, gelation^11^ or phase separation^12^ depending on the molecular interaction of the various components. By controlling the evaporation rate of the droplet and the motion of its contact line, a sequence of deposits with a rich spectrum of deposition patterns can be formed^7,13^. This evaporative self-assembly of solutes is widely used in the soft-matter, fluid-dynamics, material-science, and chemistry communities to create ordered structures on the nano- and micrometer scale, i.e. where direct manipulation is impossible^7,13–16^. In earlier phenomenological reports^17,18^, experiments revealed that remarkable well-ordered deposition patterns form by simply letting droplets consisting of a solution of collagen type I triple helices evaporate on a glass substrate. In a recent study this method has also been applied to radially orient skeletal muscle cells^19^. However, here we show a wider variety of collagen patterns to which tenocytes respond differently. The method has not yet been exploited to generate collagen platforms for the study of the physiological response of cells to different topographies and interfaces.

In this study, we present a robust and simple approach to generate complex collagen topographies consisting of isotropic and two kinds of anisotropic domains of different structures by evaporating collagen type I solution droplets. We demonstrate how the initial collagen concentration and evaporation rate of the droplet can be used to control the pattern morphology. Moreover, we explore the ability of different isotropic and anisotropic collagen patterns to steer cellular functions such as cell alignment, distribution, shape, and proliferation.

## Results

### Multiscale well-ordered organization of collagen patterns depends on concentration and humidity

**Figure 1:**
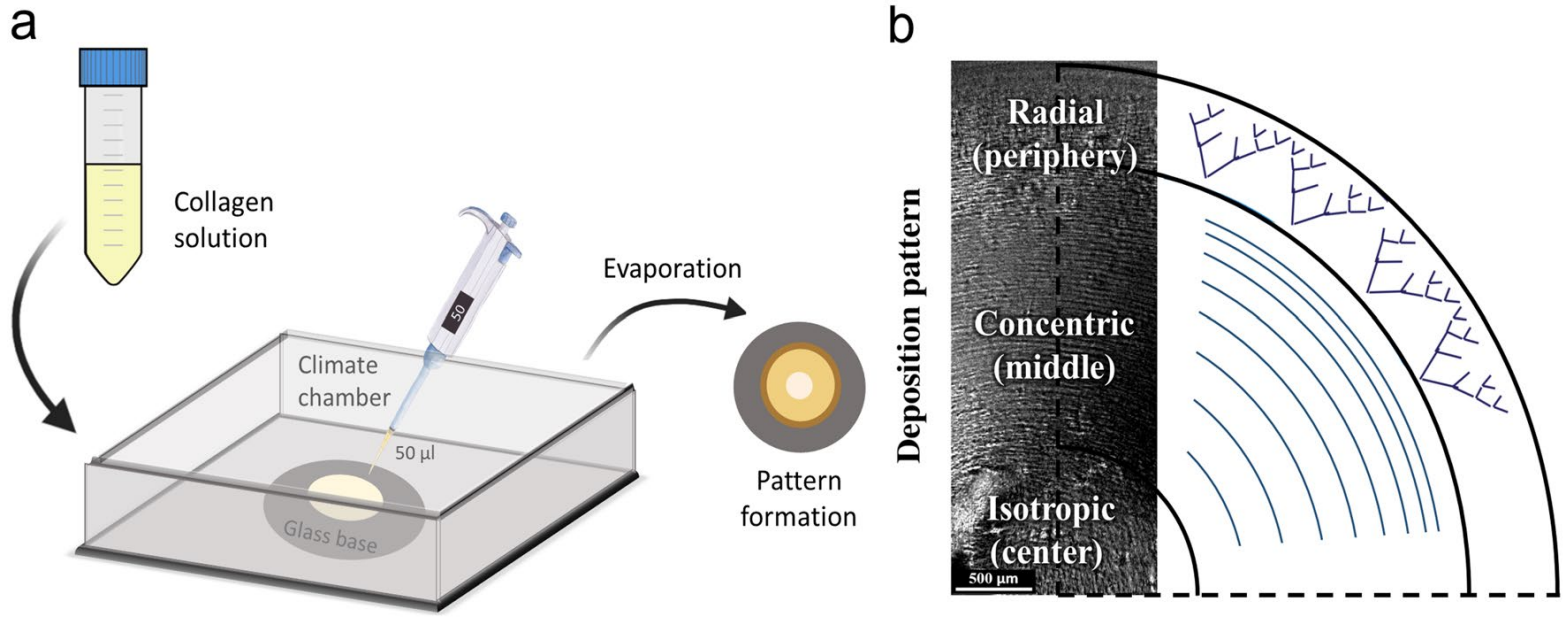
Different topographical patterns formed after collagen droplet evaporation. (**a**) Schematic overview of the climate chamber and pipetted collagen droplets on glass samples, which form a collagen stain. (**b**) Polarized light microscopy (PLM) image accompanied by a sketch of the collagen patterns formed when using a 5 mg/ml collagen solution. Three different regions formed, i.e. an isotropic region in the center, a region with concentric collagen rings patterns in the middle and a radial v-shaped pattern at the periphery. For high magnification images of the patterns, see Fig. 6b1, c1 and d1.

**Figure 2:**
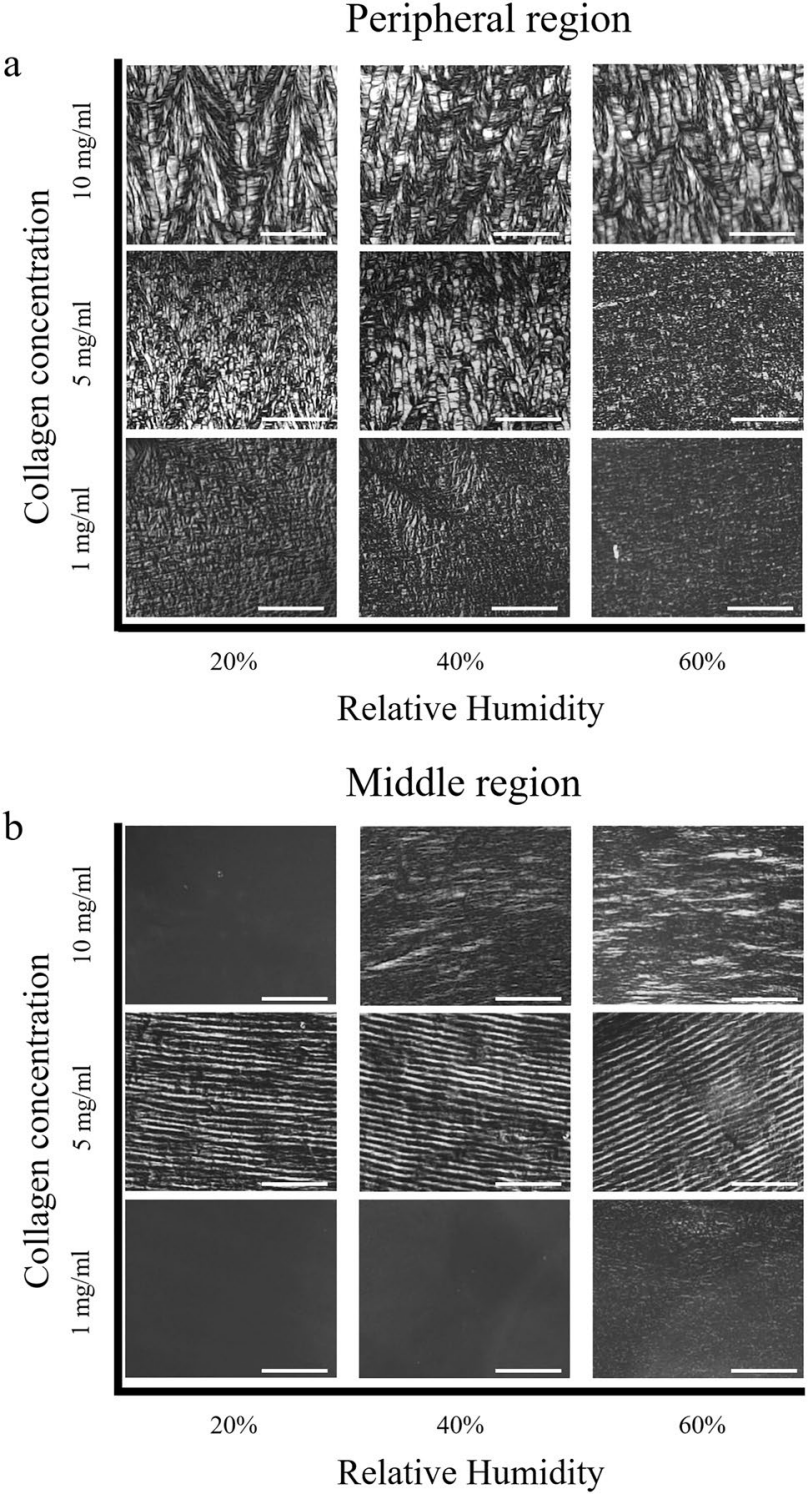
Collagen concentration and relative humidity affect the final structure of collagen stains. (**a**) Peripheral region; A radially-oriented v-shaped collagen pattern is observed at collagen concentrations of 5 mg/ml and 10 mg/ml, whereas at 1 mg/ml radial patterns were not observed. (**b**) Middle region; Concentric-ring patterns were exclusively observed at a concentration of 5 mg/ml, regardless of the relative humidity (middle row). Scale bars are 100 µm. For all experiments, *N* = 3. Displayed figures are representative images.

In this study, droplets of a collagen solution with different concentrations were evaporated on glass substrates inside a climate chamber with well-controlled humidity, schematically shown in Fig. 1a. Using Polarized Light Microscopy (PLM), we analysed the deposition patterns formed for different initial collagen concentrations and relative humidity (and hence the evaporation rate of the droplet). Three types of deposition patterns were observed. Figure 1 shows the different patterns found at the center, middle, and periphery of the dried stain. Due to the structural differences in the collagen deposition pattern in each region, as discussed below, we from now on define the regions as isotropic for the center, concentric for the middle region and radial for the periphery. A complete overview of the collagen patterns obtained at the periphery and middle regions and the influence of the relative humidity and collagen concentration is presented in Fig. 2. PLM images obtained from the center region are not included in Fig. 2 as the center region demonstrates more or less a flat homogenous characteristic. In the peripheral region radially oriented v-shaped collagen patterns appear, which becomes more abundant at high initial collagen concentration (Fig. 2a). Moving radially inwards from the periphery towards the center, different collagen patterns appeared (Fig. 2b). For collagen solutions with initial concentrations of 1 mg/ml and 10 mg/ml, little or no collagen patterning was observed in the middle region. Note that PLM only produces a signal when there is alignment of collagen that results in birefringence, i.e. polarization of the light. By consequence, the absence of a signal does not imply that there is no collagen present on the substrate.

However, well-ordered concentric-ring shaped collagen patterns were found for collagen solutions with an initial concentration of 5 mg/ml (Fig. 2b, middle row). Irrespective of the concentration or humidity, the central area consisted of a random collagen pattern. In the remainder of the paper, we will refer to the center of the stain as ‘isotropic region’, the middle of the stain as ‘concentric region’, and the periphery as ‘radial region’.

The most striking pattern formation was thus observed at an initial collagen concentration of 5 mg/ml, regardless of the humidity. A more detailed structural analysis was employed on samples that originated from an initial collagen concentration of 5 mg/ml and 20% humidity (i.e. the fastest evaporation), as shown in Fig. 2. From the periphery towards the center, initially a region of collagen molecules with a radially-oriented v-shaped organization was observed, covering only a narrow area. This peripheral region transitions into a well-ordered concentric ring pattern of collagen molecules. Finally, in the center of the drying stain an isotropic region, which lacks ordered aggregation of collagen, was found. The alternating dark and bright arcs in the concentric zone shown in the PLM image in Fig. 2 indicate a change in orientation of the collagen molecules from one ring to the next.

**Figure 3:**
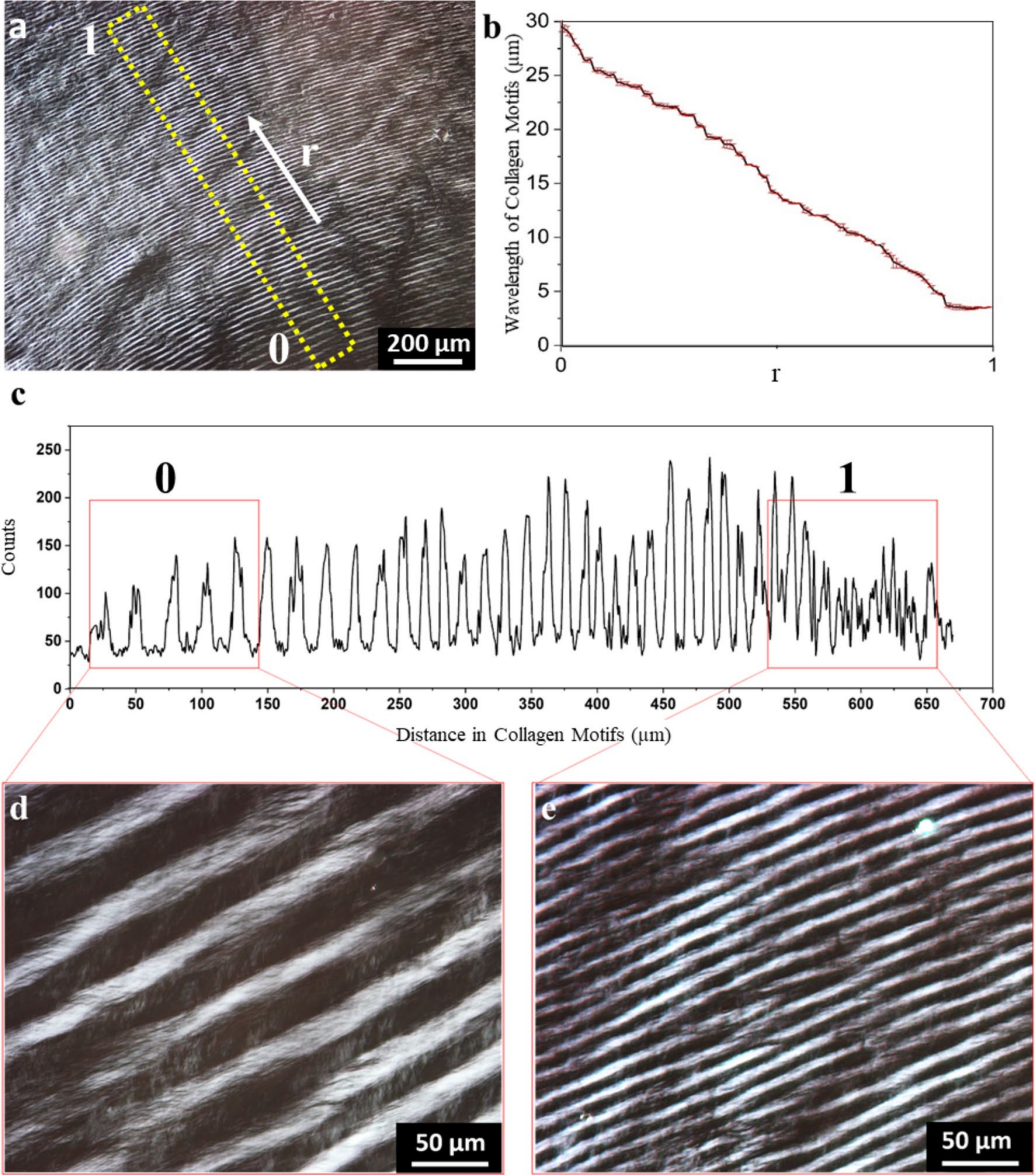
Concentric collagen patterns demonstrate differences in wavelength of collagen motifs (5 mg/ml collagen solution dried at 20% relative humidity). (**a**) PLM image of a concentric collagen pattern, the white arrow points radially outward. (**b**) The mean average of three different measurements that demonstrate the decreasing linear trend of collagen wavelength. (**c**) Wavelength of collagen patterns from the region that was highlighted with the yellow rectangle in a. (**d**, **e**) Close-up PLM images from collagen patterns that correspond to the regions 0 and 1. For all experiments, *N* = 3. Displayed figures are representative images. Error bars in b indicate the standard deviation of the mean.

Strikingly, the distance between individual concentric collagen patterns in the middle region appeared to widen moving from the periphery towards the center. To evaluate the uniformity of concentric collagen patterns we measure the distance between black and white arcs, i.e. the wavelength of the pattern, as shown in Fig. 3a. Irrespective of the concentric pattern wavelength, the alternating bright and dark collagen pattern on PLM suggests alternating orientations of the collagen fibrils within the pattern. Figure 3b,c show that the wavelength of collagen patterns indeed becomes wider upon moving radially inwards from the periphery to the center. The wavelength of the collagen patterns close to the periphery of the stain was 5.1 ± 0.9 µm while close to the center it was 26.7 ± 1.4 µm. PLM images acquired at higher magnification confirm this large difference in wavelength of the concentric rings from the periphery to center (Fig. 3d,e).

Given the results, in the remainder of the manuscript we have used 5 mg/ml collagen solutions dried at 20% humidity, which resulted in three distinct regions, namely with a radial collagen orientation at the periphery, concentric in the middle region and an isotropic orientation in the center of the stain.

**Figure 4:**
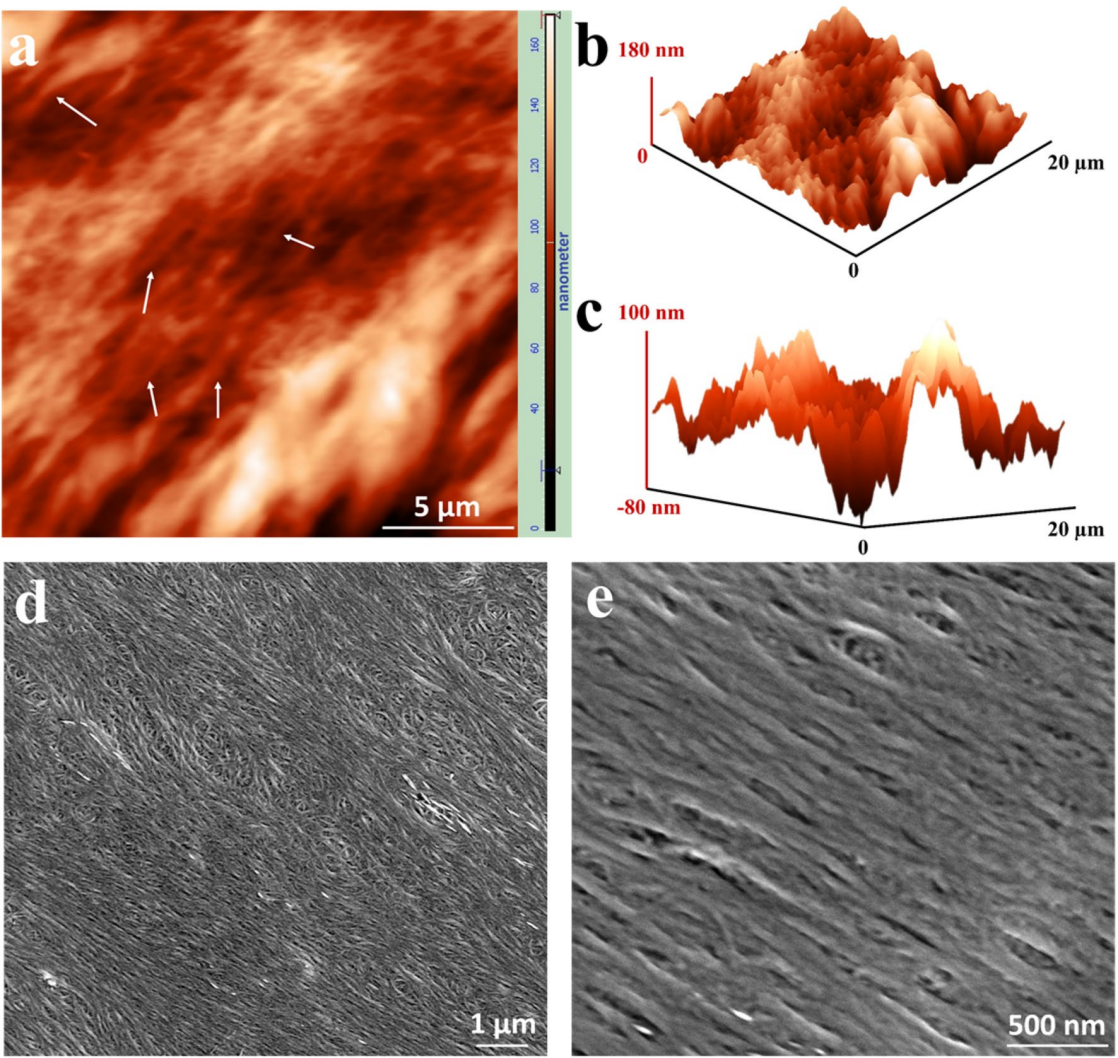
Collagen fibril formation after evaporation of a collagen droplet (5 mg/ml collagen solution at 20% relative humidity). (**a**) Atomic force microscopy image displaying the height difference of adjacent concentric patterns. Colours correspond to different heights according to the colour bar. The white arrows indicate collagen fibrils formed after drying. (**b**, **c**) Three-dimensional views of the same image in (**a**). (**d**, **e**) Scanning electron microscopy images demonstrating tightly packed collagen fibrils. Collagen fibrils of approximately 40 nm in diameter can be seen in the magnified image (**e**). For all experiments, *N* = 3*.* Displayed figures are representative images.

To further elucidate the fine details of the patterns at the nano-scale, the concentric collagen patterns were examined by atomic force microscopy (AFM) and scanning electron microscopy (SEM). From the AFM images, we observe that the alternating dark and bright regions of the concentric pattern observed under the PLM represent a valley-ridge-like topography (Fig. 4a,b,c). The height profile from two adjacent collagen patterns showed a height difference between the valley and ridge of about 180 nm (Fig. 4b,c). Moreover, Fig. 4a demonstrates fibrillar structures (marked with white arrows) which were further imaged by SEM. Figure 4d,e show that the deposition patterns consist of collagen fibrils, despite the fact that fibrillogenesis was not actively induced in our samples. SEM results confirm the presence of fibrils, which are measured as 37.7 ± 7.9 nm in diameter, by demonstrating the tightly packed collagen fibrils within the concentric pattern. Hence, we conclude that the evaporative aggregation of collagen naturally induced fibril formation within the deposition pattern.

### Pattern formation is essential for driving tenocyte alignment

**Figure 5:**
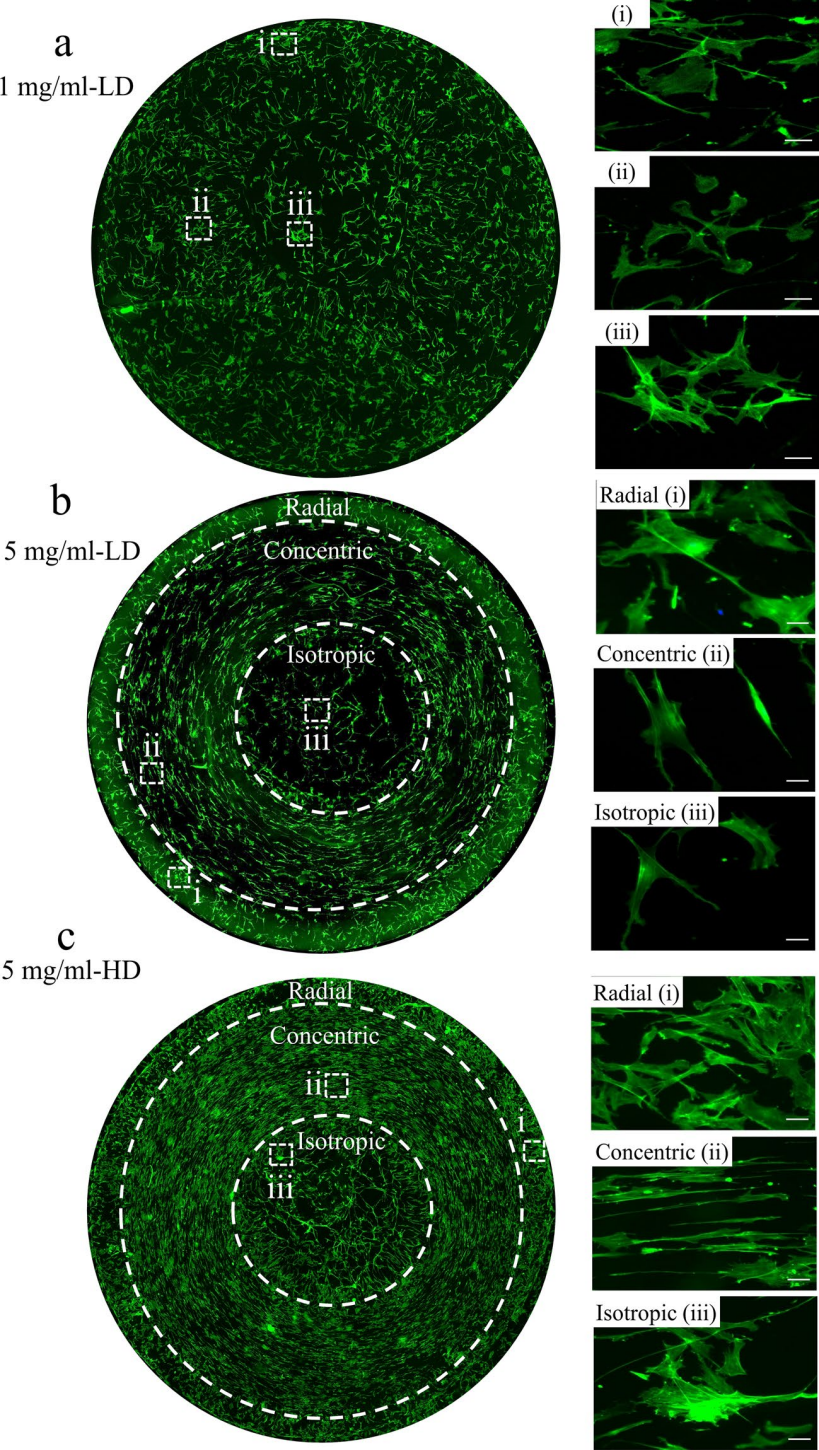
Tenocytes obey topographies created by evaporated collagen droplets. (**a**) Rat tenocytes cultured at low seeding density on 1 mg/ml collagen stains for 24 h and stained with phalloidin (green) to visualize cytoskeleton. Cells neither display any orientation, nor adapt their cell shape. (**b**) Rat tenocytes cultured at low seeding density on 5 mg/ml collagen stain for 24 h and stained with phalloidin (green) to visualize cytoskeleton. Cells display an elongated shape and a circumferential orientation aligned with the concentric pattern. (**c**) Rat tenocytes cultured at high seeding density on 5 mg/ml collagen stain for 24 h and stained with Phalloidin (green) to visualize cytoskeleton. Similar to (**b**), cells display an elongated shape and a circumferential orientation aligned with the concentric pattern. The dashed squares indicate the regions where the magnified images were captured. For all experiments, *N* = 3*.* Displayed figures are representative images.

Next, we assess whether different collagen topographies formed by evaporating droplets can promote different cell alignment. To this end, we investigate the cell response to these patterns by visualizing and quantifying cell alignment. Collagen stains were formed by evaporating droplets of 1 mg/ml (no distinct pattern formation) and 5 mg/ml collagen solution at 20% humidity (3 distinct patterns). Rat tenocytes were seeded at low density (LD, 1000 cells/cm^2^), fixed after 24 h, and stained with Phalloidin (green) to visualize the actin cytoskeleton (Fig. 5). In absence of a clear pattern formation at 1 mg/ml, cells were randomly distributed and did not display differences in cell morphology between locations (Fig. 5a and supplementary Fig. 1). For the 5 mg/ml samples, where clear collagen patterns were observed, we first identified the regions that cells attach to by using PLM. Subsequently, cellular alignment was assessed: i.e. cells aligned preferentially radially in the radial region, strongly circumferentially with a spindle-shaped morphology in the concentric region and randomly in the central isotropic region (Fig. 5b).

Previous research has shown that cell density influences cell shape, migration and downstream signalling^18^, and may possibly override the effect of the substrate topography. Therefore, we investigated whether the observed influence of topography on cell shape in a confluent cell culture is still effective. To this end, rat tenocytes were seeded at high density (HD, 10,000 cells/cm^2^) and visualized as explained earlier (Fig. 5c). Despite confluency, cells exposed to the pattern still displayed a strong alignment and spindle-shaped morphology with the patterns they were exposed to (Fig. 5c). The different wavelengths of the concentric-ring topographies (as displayed in Fig. 3 and supplementary Fig. 2) in the concentric – middle region did not appear to affect cell alignment, in both LD and HD conditions. Overall, our results indicate that the concentric ring pattern in the 5 mg/ml-stain can promote cellular orientation and morphology, regardless of the cell-seeding density.

### Tenocyte orientation and shape are altered by different topographies

**Figure 6:**
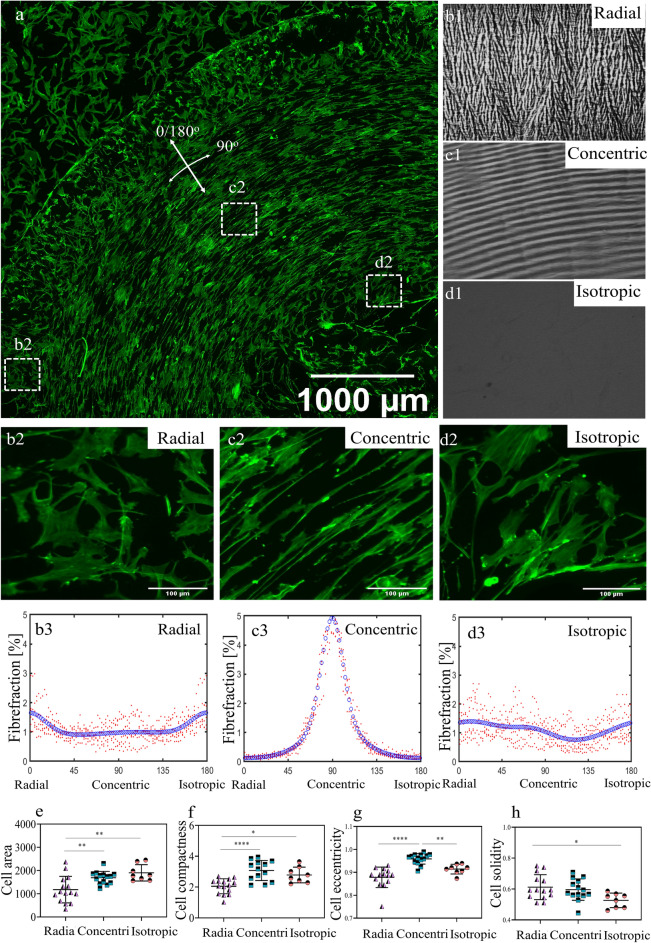
Topographies created after evaporation of collagen droplets influence tenocyte orientation and shape. (**a**) Quarter of a collagen stain that is seeded with Phalloidin-stained rat tenocytes (5 mg/ml collagen concentration, cells seeded at high density). (**b**1, **c**1 and **d**1) Representative image of the radial, concentric and random region, respectively. (**b**2, **c**2 and **d**2) Representative higher magnification phalloidin images. (**b**3, **c**3 and **d**3) F-actin fiber distributions showing a radial, concentric and random fiber distribution for the three respective regions. Red dots represent individual fiber fractions of the images analysed, where the blue line represents a bimodal fit. *R*^2^ values of b3, c3 and d3 are 0.31, 0.97 and 0.19, respectively. (**e**) Quantification of cell area, (**f**) eccentricity, (**g**) compactness and (**h**) solidity for cells on radial, concentric and isotropic regions. Scale bars in b2-d2 represent 100 µm. In e–h, each symbol represents a single image. Area, eccentricity, solidity and compactness values are represented in arbitrary units. Error bars represent 95% confidence intervals. Each asterisk represents statistical significance of the differences of cell area, compactness, eccentricity and solidity in redial, concentric and isotropic regions. *P < 0.05, **P < 0.01. For all experiments, *N* = 5. The dashed squares indicate the regions where the magnified images were captured.

To further assess the strength of the pattern in driving cellular alignment and morphology, cellular F-actin was visualized and quantified (Fig. 6). Results reveal that the F-actin orientation in the concentric region displayed a clear peak at 90° , representative for a strong collagen anisotropy in the concentric direction (Fig. 6.b1-b3). In the radial region, collagen displayed a marginal preference for the 0/180° angle, representative for collagen to align to the radial direction (Fig. 6.c1-c3). In this region, we did not observe a clear cell alignment based on both visual inspection (Fig. 6b2) and alignment calculations (Fig. 6b3), yet only a small preference towards the radial direction was observed. In the isotropic region, no preferred collagen orientation was found, since the bimodal fit (represented by the blue dotted line) only has an R^2^ of 0.19 (Fig. 6.d1-d3). Therefore, despite that, the collagen topographies strongly differed between the radial and central regions according to the polarized light microscopy images, they failed to induce a striking difference in cellular alignment.

Subsequently, cell shape was quantified in terms of area, eccentricity, compactness, and solidity (Fig. 6) and performed ANOVA to determine the statistical difference. Cells located on the radial region displayed the smallest cell area and cells in the isotropic region the largest cell area. Cellular eccentricity, a measure for cell elongation (the more elongated the cell, the closest the eccentricity value is to 1), was highest for cells in the concentric region. Despite the modest anisotropic orientation of the cells in the radial region, the eccentricity was lower compared to the random region (Fig. 6f). Cell compactness (higher value indicates a more elongated cell) was highest for cells on the concentric region (Fig. 6g). Cell solidity (values are oppositely correlated with cell branching and filopodial protrusions) was highest for cells on the radial region and significantly different compared to cells in the isotropic region (Fig. 6h). Overall, these results indicate that concentric regions induced aligned and more elongated, i.e. spindle-shaped, tenocyte morphology, whereas isotropic and radial regions only induced differences in cellular morphology.

### Topographical architectures on collagen stains affect cell density

**Figure 7:**
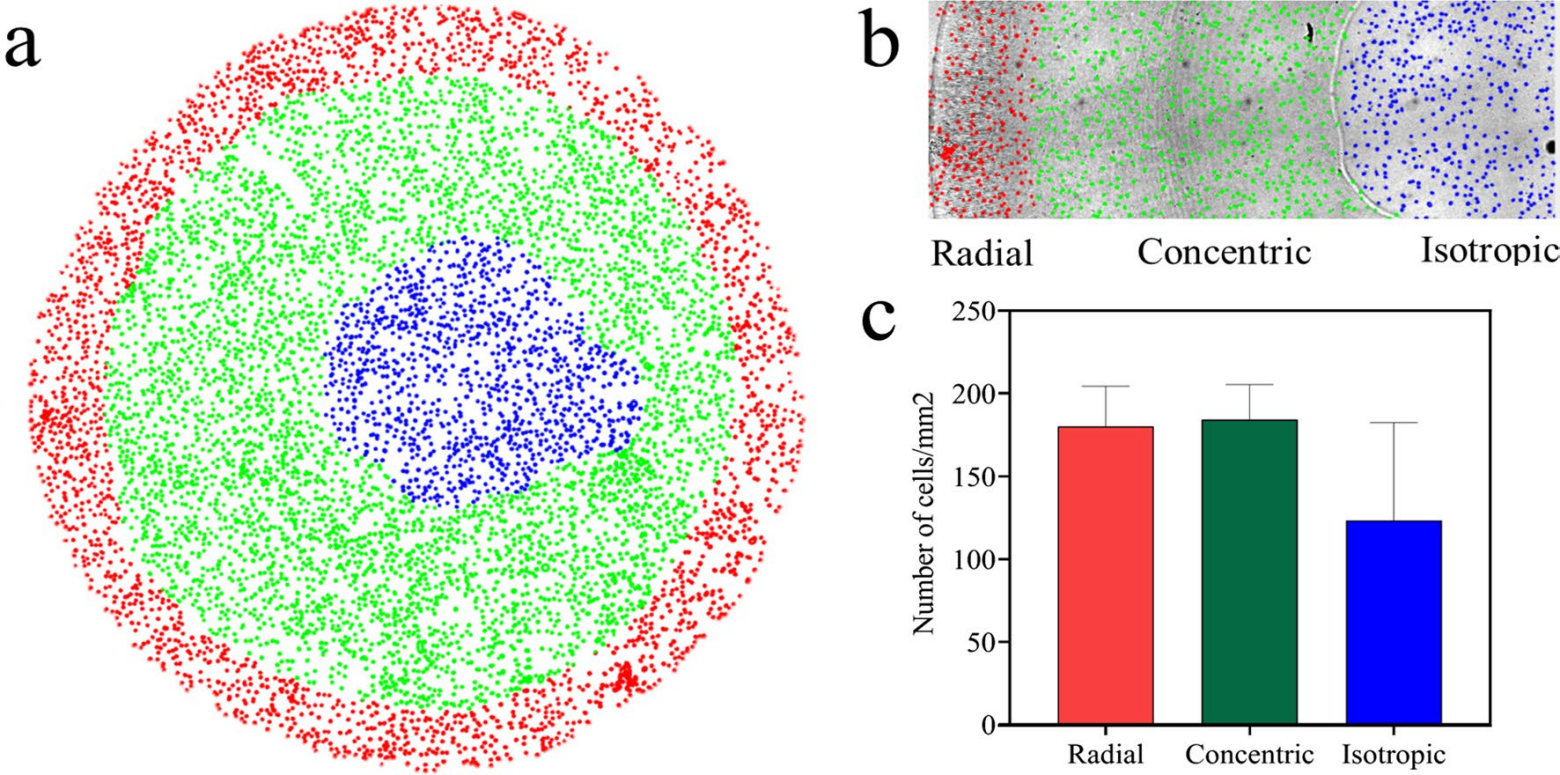
Cell density varies between patterns and is lowest for the isotropic region. (**a**) The cells are labelled red, green and blue for radial, concentric and isotropic regions. (**b**) Detailed view of the cells (with the same colour coding as in a) together with the corresponding stain, shown in the background. (**c**) The average densities in the radial (180 ± 24), concentric (184 ± 21) and isotropic region 123.3 ± 59 cells/mm^2^ are displayed. Error bars represent 95% confidence intervals. For all experiments, *N* = 3.

Surface topography is known to affect not only cell shape, but also proliferation and differentiation^18,20^. Therefore, as a next step we investigated how the different topographies of the collagen stains affected the cell density as illustrated in Fig. 7. The different colours of the cells indicate the three regions. To obtain the average cell densities we calculated the total number of cells per region and the corresponding area of that region (Fig. 7a, b). After performing ANOVA test, our results show that on average the cell density is 180 ± 24 cells/mm^2^ in the radial region, 184 ± 21 in the concentric region and 123.3 ± 59 cells/mm^2^ in the isotropic region (Fig. 7c). This results suggest that there is no significant difference in cell density between radial, concentric and isotropic region.

### Surface topography does not alter cell proliferation

**Figure 8:**
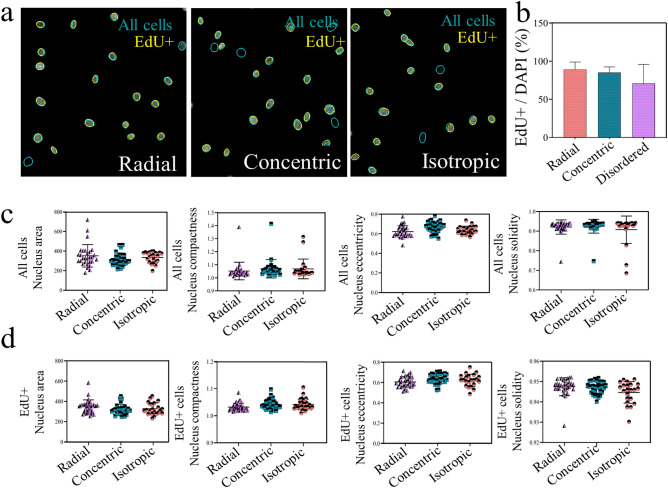
Tenocyte proliferation and nucleus shape was not affected by topographies. (**a**) Representative image of all cells (DAPI, Green–Blue) and EdU positive cells (yellow) on radial, concentric and isotropic regions. (**b**) Quantification of EdU positive cells with a trend towards lower proliferation in the isotropic region. (**c**) Quantification of nuclear shape parameters on the different regions for all cells (area, compactness, eccentricity, solidity) and (**c**) for EdU^+^ cells. Each symbol represents the average of a single image. Error bars represent 95% confidence intervals. For all experiments, *N* = 3.

Cell proliferation is a feature, known to be affected by environmental factors, including surface topography^21^. Concordantly, we assessed whether the topography-induced cell shape results in different proliferation rates after 24 h of seeding (Fig. 8). Image analysis showed that in isotropic region 70 ± 25%, in radial region 89 ± 9% and in concentric region 85 ± 7% of the cells were EdU positive, hence proliferating, and no statistical difference detected. (Fig. 8b). As shown in our previous work^22^, a surface topography that alters cellular morphology (as found in the radial and concentric zones, see Fig. 6a) can result in a decrease in nuclear size, and hence a decrease in cell proliferation^22^. Therefore, we further evaluated whether topographies on the radial, concentric and isotropic region induce changes in the nucleus shape in all cells and also specifically (Fig. 8c) EdU positive cells (proliferating cells) (Fig. 8d). The nucleus area, compactness, elongation, and solidity were similar for all regions, and did not differ between EdU positive cells (Fig. 8 c&d). These results indicate that proliferation and nucleus shape remained unaffected by the surface topographies.

## Discussion and conclusion

In the present work, we demonstrated a simple method to create multiscale well-ordered collagen patterns by evaporating a droplet containing collagen molecules in a well-controlled environment and investigated how cells are affected upon being cultured on this material.

The final deposition pattern that forms is the result of a complex interplay between the evaporation-driven capillary flow inside the droplet^23,24^ that transports the collagen fibers towards the contact line, the motion and/or pinning of the contact line^13,25^, the interaction between the collagen and the substrate^26^ and the solution rheology^27^. At the periphery of the stain, a pattern of radially-oriented v-shaped stripes was observed. These structures are the first that form as the droplet evaporates. Similar deposits for colloidal-particle laden droplets have been reported^28^. Possible explanations for these structures range from buckling caused by the build-up of internal stress during desiccation, shear banding due to internal flow^29^, and contact line instabilities^30^, but the precise mechanism remains to be explored. Further experiments are necessary to reveal whether the radial deposit forms during the pinning phase of the contact line, and hence result from the self-assembly of the collagen fibers upon accumulation at the contact line by the capillary flow^23^, or during the first stages of contact line recession. In later stages of the droplet life, when the contact line recedes while the contact angle remains constant, regular concentric ring patterns were formed consisting of ridges and valleys. In literature, such robust periodic patterns have been reported for evaporating polymer solutions^13^ and are attributed to a stick–slip motion of the contact line that is caused by pinning/depinning events^31^. In polymer solutions, the regularity of the pattern is caused by a self-organized pinning-depinning cycle that results from local changes in the solution viscosity due to the convective transport of material to the contact line^31^. A detailed analysis of the collagen aggregation in the radial and concentric regions in presence of evaporation-driven capillary flow and contact-line motion is left for future work. Clearly, there is a need for a full exploration of the parameter space and the development of adequate models to predict the patterns that will form and when pattern-to-pattern transitions will occur, depending on e.g. the droplet volume, evaporation rate and collagen concentration. Such modelling will help to delineate the differences between the results of Nerger et al.^19^, where only radial patterns are found, and the present work where both radial and concentric ring patterns reproducibly formed. Furthermore, it is not so clear why a concentration of 5 mg/ml yields a much more pounced topography than either 1 mg/ml or 10 mg/ml. The useful concentration window seems to be rather narrow, but the process probably can be optimised. Additionally, the transition from concentric to isotropic collagen organisation that occurs during the final stage of the droplet life needs to be further investigated to determine the influence of e.g. gelation^11^, rapid solute accumulation causing order-to-disorder transitions^23^ and collagen adhesion on the absence of a well-defined pattern anisotropy in the center of the stain.

Studying the interactions between cells and their surrounding extracellular matrix is one of the main steps on the path to understanding mechanisms of health and disease; hence, here we investigated how the nano-topographies created upon self-assembly of collagen molecules affect tenocytes with respect to their cytoskeletal organization, orientation and proliferation. We first evaluated the impact on topography on cell shape as upon cell attachment, initial changes are observed in the cell cytoskeleton. Different cell types (e.g. mesenchymal stem cells, tenocytes, epithelial cells) have been shown to adopt their cytoskeleton based on the micro-topographies^22,32–34^ and nano-topographies^35,36^ that they exposed to. The topographies on the collagen stains are at nano-level and forced tenocytes to form an elongated shape. This result is also supported by Yang et al. where they show that nano topographies as small as 50 nm led elongation and orientation of human dermal fibroblasts^37^, and human bone marrow-derived mesenchymal stem cells^38^. However, characteristics (pitch, ridge, and groove dimensions) of the nano-groove surface topography matter and can either result in elongated cell shape or lead to the formation of spherical aggregates of human tenocytes^39^.

In the current study, we used rat tenocytes as a cell type to investigate the role of surface topography on cell behaviour for the following reasons: 1) Tendon tissue naturally possesses a hierarchical order of strongly anisotropic collagen fibers. 2) Tenocytes naturally possess an elongated shape in the native tissue. However, upon culturing tenocytes on a flat surface, or upon disruption of the native tissue architecture, they dedifferentiate, change their morphology to become more spread, alter their phenotypical marker expression, and proliferation characteristics. 3) In the native tendon, tenocytes experience biomechanical cues originating from collagen fibrils at the nanometer scale (20–150 nm) and fibers are at micrometer-scale (10–50 µm)^40^, suggesting that they can respond to very small topographies, such as the concentric rings. Concentric rings in the collagen stain, which have 180 nm depth valleys in between, pushed tenocytes to an elongated morphology, which is characterized by an eccentricity value close to 1. Additionally, in the native tendon, tenocytes are oriented in the direction of collagen fibers, which was also accomplished by concentric rings. Furthermore, since formation of hierarchical aggregation of collagen is challenging in an in vitro system, our system allows tenocytes to experience more “*natural*” environment, particularly in the concentric ring with respect to topographical architecture and substrate itself and hence tenocytes are exposed to a rather natural ECM that is closed to a native healthy tendon. Therefore, collagen stains containing these concentric rings are excellent tools to give the sense of a natural ECM to tenocytes and further investigate cell-biomaterial interactions with respect to tenocyte biology.

Proliferation is one of the highly investigated read-outs on cell-biomaterial interaction studies as in vivo cells do respond to changes in tissue architecture. For instance, in the heathy tendon, tenocytes are relatively quiescent and start proliferating during tissue remodelling in case the tissue anisotropy is lost^41,42^. This in vivo phenomenon was recently replicated in *vitro* by showing that, upon culturing tenocytes^32^ and human mesenchymal stem cells^43^ on a tendon-biomimetic topography which possess anisotropic topographical features, tenocytes displayed a quiescent phenotype compared to cells cultured on a flat surface. Therefore, we investigated cell-biomaterial interactions with respect to cell density and proliferation in order to elaborate on the influence of isotropy or anisotropy in analogy to healthy and damaged tissue. To our surprise, we did not observe striking differences in cell proliferation between radial, concentric and isotropic regions, similar to the cell density after 24 h. It is hypothesized that the nuclear deformation can lead to changes in chromatin structure and therefore led to a decrease in cell proliferation^22,44^. For this reason, we calculated the deformation in the nucleus with respect to its shape but did not observe differences between regions or between EdU negative and positive cells. In a future study, we will focus on how to control the wavelength, width, and height of the concentric ring pattern, to investigate the possibility to optimize the pattern for inducing nuclear shape changes/cell proliferation. It was also suggested by the work of Milner and Siedlecki et al.^45^ and Christopherson *et. al*.^46^ that a reduction in feature sizes on the material eradicated the discrepancy between nano-topographies and flat substrates in terms of cell proliferation. Similar to our proliferation results, we did not observe any significant differences in cell density after 24 h between the different regions. However, our experiments suggest a decrease in cell density in the isotropic region compared to the concentric and radial regions. Future experiments are required to investigate this trend, and delineate whether its origin lies in differences in cell migration, proliferation or apoptosis between the different zones.

Nevertheless, other mechanisms such as integrin signalling and focal adhesion dynamics also influence cell proliferation and cell adhesion. Integrin signalling is one of the first steps upon cell attachment and initiates downstream signalling cascades for cellular responses such as cell adhesion, growth, motility, shape, and differentiation^47^. Integrins are transmembrane proteins that modulate cell-biomaterial crosstalk by interacting their extracellular domain with the ECM and the intracellular domain with signalling molecules and can be activated by both surface topography^48–50^ and surface chemistry^51–53^. However, it is still under debate whether cells react initially to surface chemistry^54,55^ or surface topograpy^55,56^. In evaporated collagen, all the regions are formed by collagen, and therefore the ligand possibly suppresses the effect of topographical differences. As a result, cell proliferation was not significantly different between regions and between cells. To support this, proliferation of human osteoblasts on nano rough titanium films was similar to that on smooth surfaces^57^. Furthermore, proliferation of human mesenchymal stem cells on nano topographic poly(methyl methacrylate) (PMMA) was shown to be increased after 14 days of culture compared to their smooth counterparts^58^. Accordingly, we suggest two possible explanations for our proliferation results: 1) The height difference between the valley and ridge, providing the topographical cue, is too low to cause a nuclear deformation, and thus do not strongly affect proliferation rate. This is supported by the lack of any topography on the isotropic region, which even displayed a decreasing trend in the proliferation. 2) Collagen as a substrate that cells bind to create a stronger signal compared to the topography of that collagen, and therefore the nuclear response towards proliferation was fairly similar. Nonetheless, considering that surface topography and chemistry act together to produce a biological response, these results should be further explored. For instance, focal adhesion kinase (FAK), Src, and Rho GTPases are among the downstream effectors of the integrin signalling that are known to affect cell proliferation as well as cytoskeletal changes^50^, hence, further investigation on the activation of these pathways can aid us to understand the effect of integrin signalling on the proliferation.

The topographies created by the evaporation of collagen droplets enables studying cell-biomaterial interactions. Firstly, the stains consist of three different topographical regions and with sharp interfaces in between, which enables a direct comparison of read-outs in the same sample. Firstly, how cells migrate on different topographies, a cell's preference to migrate towards certain topography and cell behaviour at the interface between different topographies can be investigated within a single sample. Secondly, topographies are made up of collagen type I, which is one of the most abundant types of collagen in the connective tissues such as bone and tendon. Therefore, together with biomechanical cues created by topographies, the effect of the biochemical cues can be investigated.

Overall, our robust and simple approach to generate complex topographical collagen patterns by droplet evaporation can be used to unravel cell–matrix interactions and better understand and control cellular function. The collagen stains formed bear three distinct patterns and their interfaces, which can mimic physiological conditions. For example, the concentric pattern can be used to mimic a healthy tendon and the isotropic region to feature a tendinopathic condition. Thereby, our material can be used to reveal how cells respond to the different patterns and their interfaces in terms of cellular capacity for functional remodeling and to explore strategies to push cells on the e.g. isotropic patterns to display improved functional remodeling capacity. In addition, the underlying molecular mechanisms that affect this remodeling capacity, associated with topography-induced cell shape, can be studied. Hereby, optimal control over the pattern formed by evaporation is essential. In future work, we will therefore focus on mapping out the parameter space for collagen pattern formation inside evaporating drops and identifying the key physical mechanisms that control the pattern.

## Materials and method

### Preparation of collagen solutions

Lyophilized collagen I from calfskin (Elastin products, C857) was dissolved in 0.5 M acetic acid at 1 mg/ml, 5 mg/ ml and 10 mg/ml collagen concentrations and vortexed vigorously to ensure collagen was dissolved in the solution. Next, solutions were centrifuged 5 min at 300 RCF. For each experiment, fresh solutions were prepared.

### Climate chamber and sample preparation

The collagen stains are made by evaporating 50 μL droplets of the fresh collagen solution. Five droplets are pipetted on different glasses and placed inside the climate chamber. The chamber is a transparent plexiglas box in which the temperature is monitored and is 23 ± 1 °C for all the experiments. The relative humidity in the chamber is controlled via a N_2_-gas inflow to lower the humidity to a specified level. After an initial transient of about 15 min, a stable humidity is obtained within ± 1% of the target value (a typical experiment lasts 2.5 h). Droplet evaporation is recorded in side- and bottom-view. In the bottom-view, the formation of the stain and the contact line motion are recorded via Bright Light Microscopy (BLM). A side-view camera is used to measure the size of the droplet and the contact angle with the glass substrate over time. Once all the liquid has evaporated, the collagen stains are stored at 4 °C until use.

### Isolation of rat tenocytes

Rats were collected after euthanization considering their surplus status from the breeding program. Tenocytes were isolated from the hind limbs of 23 weeks old Cyp1a2ren strain rats by using a previously published protocol^32^. Briefly, tendons from the hind limbs were digested in a solution containing collagenase type II (3 mg/ml) (Worthington Biochemical), dispase II (Sigma-Aldrich) (4 mg/ml) and 100 U/ml Penicillin/Streptomycin (Thermo Fisher Scientific) for 4 h at 37 °C. Next, the solution was passed through a 70 mm cell strainer (Life sciences) and further spinned at 300 G for 5 min. Then, pellet was re-suspended in Dulbecco's modified Eagle's medium (DMEM, Sigma-Aldrich) supplemented with 10% fetal bovine serum (FBS), 100 U/ml penicillin/streptomycin, tenocytes were cultivated in T-25 flasks until 70% confluency.

### Sterilization of tendon imprints and cell culture

Collagen stains was sterilized by incubating the materials in 70% ethanol for 30 min. Next, ethanol was aspirated, and remaining ethanol let to air-dry under sterile conditions. Subsequently, samples were washed in sterile phosphate-buffered saline (PBS, Sigma-Aldrich) at 37 °C three times and washed with culture medium twice before use. Passage 4 tenocytes were seeded at 1000 cells/cm^2^ (for low-density culture) and 10,000 cells/cm^2^ (for high density culture) for subsequent orientation, cell density and proliferation analyses.

### Phalloidin and EdU staining

Upon 24 h after seeding, cells were fixed with 4% paraformaldehyde (PFA, ThermoFisher Scientific) at room temperature for 20 min and subsequently washed with PBS. Afterwards, samples were incubated in were incubated in Phalloidin–Tetramethylrhodamine B isothiocyanate (Phalloidin-TRITC, 1:200; ThermoFisher) in PBS for 1 h and washed 3 times with PBS to stain F-actin. Next, 4′,6-diamidino-2-phenylindole (DAPI, 1:500; Sigma-Aldrich) in PBS was used to stain nuclei for 1 h and further washed 3 times with PBS. Finally, samples were mounted on glass cover slides with mounting medium (Dako, Agilent). Samples were stored at 4 °C at dark.

Click-iT EdU Cell Proliferation Kit (Invitrogen, C10340) for Imaging (Thermo Fisher) was used to detect proliferating cells as described on the manufacturer’s instructions. Briefly, cells were serum-starved for 24 h before to EdU labelling to synchronize their biological clock. Next, cells were fixed with 4% paraformaldehyde (PFA, ThermoFisher Scientific) at room temperature for 20 min and permeabilized with 0.5% (v/v) Triton X-100 in PBS for 20 min after 24 h of incubation in 10 µM EdU solution. Afterwards, cells were treated with EdU reaction cocktail for 30 min in the dark and incubated in Hoechst for another 30 min to stain the nucleus. Next, samples were mounted on glass cover slides and stored at 4 °C at dark.

### Imaging

Borosilicate Glass substrates that were employed to dry collagen solutions, were used without any further sample preparation step. Regions of interest were imaged between two polarizers with a Zeiss Axioplan 2 light microscope using transmission mode at magnifications 10 × , 20 × , and 50 × . The scanning electron microscopy (SEM) imaging of the glass substrates was performed by using a FEI Quanta FEG 600 (Thermo Fisher Scientific). Glass substrates were sputter-coated prior to SEM imaging in order to avoid a possible charging effect and to improve the imaging quality. Atomic force microscopy (AFM) measurements were conducted by using a NTegra Agura (NT-MDT) machine in tapping mode and Si microcantilever probes with a spring constant of 5 N/m. Fluorescent images were taken with a Leica DMi8 with TIRF Multi Color microscope (Leica Microsystems CMS) at 10 × magnification. Lasers at excitation wavelengths of 532 nm and 647 nm were used for phalloidin and EdU respectively. Tile images were stitched together with LAS-AF Lite version 2.6 (Leica Microsystems CMS).

### Quantification of F-actin orientation by fiber tracking and bimodal fitting

F-actin stress-fiber orientation was measured by using actin stained samples based on previously developed fiber-tracking algorithm^54^. Briefly, via employing a multi-scale approach the principle curvature directions from the eigenvalues and the eigenvectors of the Hessian matrix of the image were calculated. For each image, a histogram that contains fiber fraction per angle (ranging from 0° to 180° and 0° to 90° degrees with a 2° interval) was obtained. For bimodal fitting, average fiber fractions for all images from the same protocol were used. To quantify the fiber distribution, the experimentally observed fractions were approximated by a bi-modal periodic normal probability distribution function using a nonlinear least-squares approximation algorithm:

$$\varphi_{f} \left( \gamma \right) = A_{1} \exp \left[ {\frac{{\cos \left( {2\left( {\gamma - \alpha_{1} } \right) + 1} \right)}}{{\beta_{1} }}} \right] + A_{2} \exp \left[ {\frac{{\cos \left( {2\left( {\gamma - \alpha_{2} } \right) + 1} \right)}}{{\beta_{2} }}} \right]$$

Hereby, ${\varphi }_{f}\left(\gamma \right)$ is the fiber fraction as a function of the fiber angle $\gamma$. Variables ${\alpha }_{1}$ and ${\alpha }_{2}$ are the two main fiber angles and ${\beta }_{1}$ and ${\beta }_{2}$ represent the dispersities of the two fiber distributions. An angle of 90° is parallel to the radial direction and aligned with the concentric direction. The parameters ${A}_{1}$ and ${A}_{2}$ are scaling factors for the total fiber fractions of the distributions.

### Image analysis

Analysis of acquired PLM and SEM images was done by using the software packages provided by Digital Gatan Micrograph and Image J such as Plot Profile tool to quantitively measure the wavelength of the collagen patterns and diameter of collagen fibrils. Approximately 60 images were used to calculate the wavelength of the collagen patterns and more than 100 collagen fibrils were picked to measure the diameter of collagen fibrils and results are reported as mean ± standard deviation of the mean. Cell and nucleus shape parameters were extracted from CellProfiler^55^ by using customized pipelines for Phalloidin and EdU analysis. Each pipeline included background correction, nucleus and cell segmentations for further cell and nucleus shape analysis. Cell shape parameters were calculated by CellProfiler: Eccentricity is calculated by taking the ratio of the distance between the foci of the ellipse and its major axis length. Compactness calculated by the mean squared distance of the object’s pixels from the centroid divided by the area. Solidity calculated by taking the proportion of the pixels in the convex hull that are also in the object^32,59^. The analysis on the regional cell densities are done with MATLAB. First, PLM images are used to define masks for the radial, concentric and isotropic region. The area and number of cells per region are calculated after placing the corresponding masks on the DAPI images. The cells in the image (white) are easily distinguished from the surrounding background (black) with a threshold in the grayscale values. Graphs are drown in GraphPad Prism version 8.0 (GraphPad Software, Inc., San Diego, CA) and images are prepared in Fiji^60^.

### Statistics

Statistical analyses for cell and nucleus shape analysis and proliferation were performed by using GraphPad Prism version 8.0 (GraphPad Software, Inc., San Diego, CA). One-way analysis of variance (ANOVA) was carried out to calculate the statistical difference in three different regions. For all statistical analysis, significance is set at *p* < 0.05 and significance was determined by Tukey’s post-hoc test.

## Supplementary information

Supplementary Figures.
