# Supplementary material for: Self-agglomerated collagen patterns govern cell behaviour
Source: Sci Rep. 2021 Jan 15;11:1516. doi: 10.1038/s41598-021-81054-5 (PMC7810981; doi:10.1038/s41598-021-81054-5)
Supplement: Supplementary file 1 — Supplementary Figures. [file 41598_2021_81054_MOESM1_ESM.docx]

# **Self-agglomerated collagen patterns govern cell behaviour**

Aysegul Dede Eren^1,5 †,^, E. Deniz Eren^2†^, Twan J.S. Wilting^3†,^, Jan de Boer^1,5^, Hanneke Gelderblom^3^***, and Jasper Foolen^4,5^***

^1^Biointerface Science group, Institute for Complex Molecular Systems, Department of Biomedical Engineering, Eindhoven University of Technology, Eindhoven, The Netherlands.

^2^Laboratory of Physical Chemistry, Department of Chemical Engineering and Chemistry, Eindhoven University of Technology, Eindhoven, The Netherlands

^3^Fluids and Flows group, J.M. Burgers Centre for Fluid Dynamics, Department of Applied Physics, Eindhoven University of Technology, Eindhoven, The Netherlands

^4^Regenerative Engineering & Materials, Department of Biomedical Engineering, Eindhoven University of Technology, Eindhoven, The Netherlands

^5^Institute of Complex Molecular Systems, Eindhoven University of Technology, Eindhoven, The Netherlands

^†^*These authors contributed equally to this work*

**Correspondence to:* [*h.gelderblom@tue.nl*](mailto:h.gelderblom@tue.nl)*,* [*jfoolen@tue.nl*](mailto:jfoolen@tue.nl)


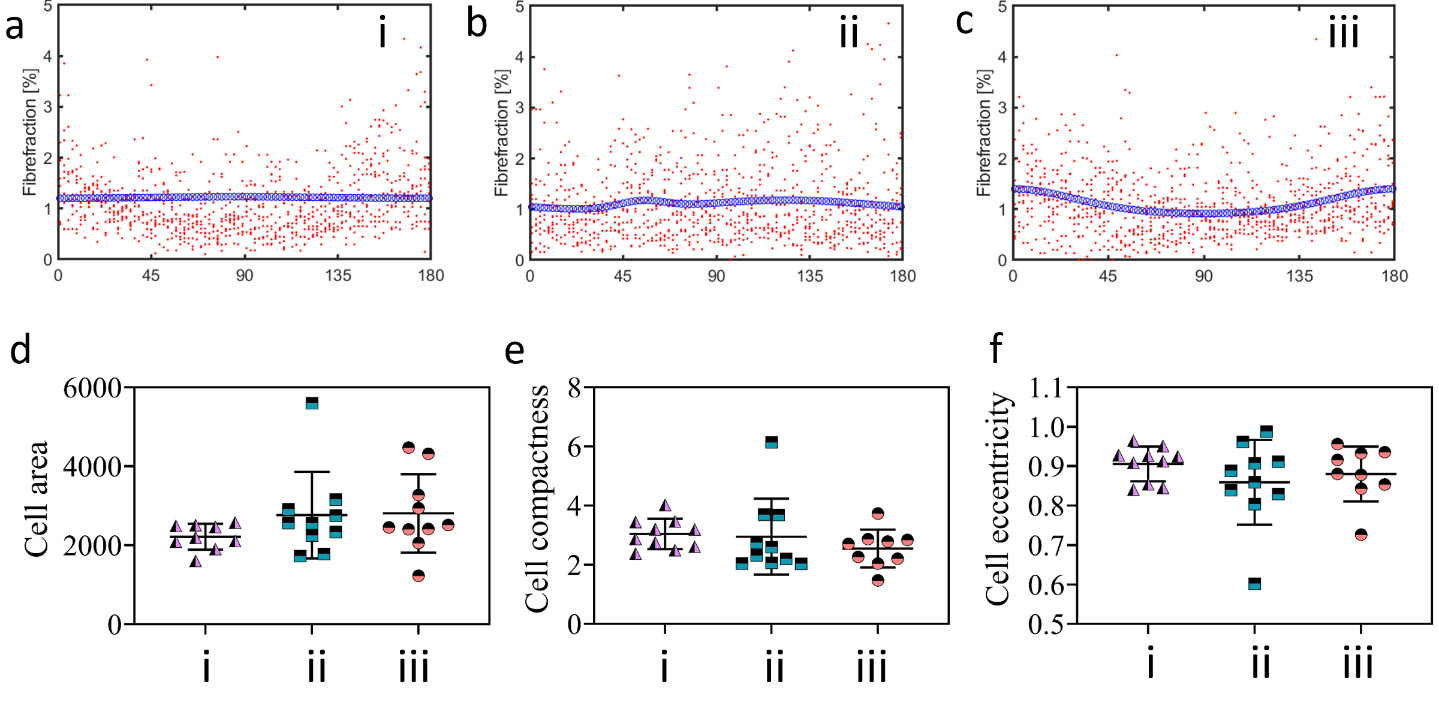


**Supplementary Figure 1.** Random cellular alignment on a collagen stain, derived from evaporating a 1mg/ml collagen droplet. Images are obtained from randomly selected regions close to the stain in (i) the peripheral region, (ii) the middle region, and (iii) the central region were quantified for orientation and cell morphological parameters. Tenocytes neither displayed preferred orientations (a-c), nor differences in cell shape, between locations (d-f).


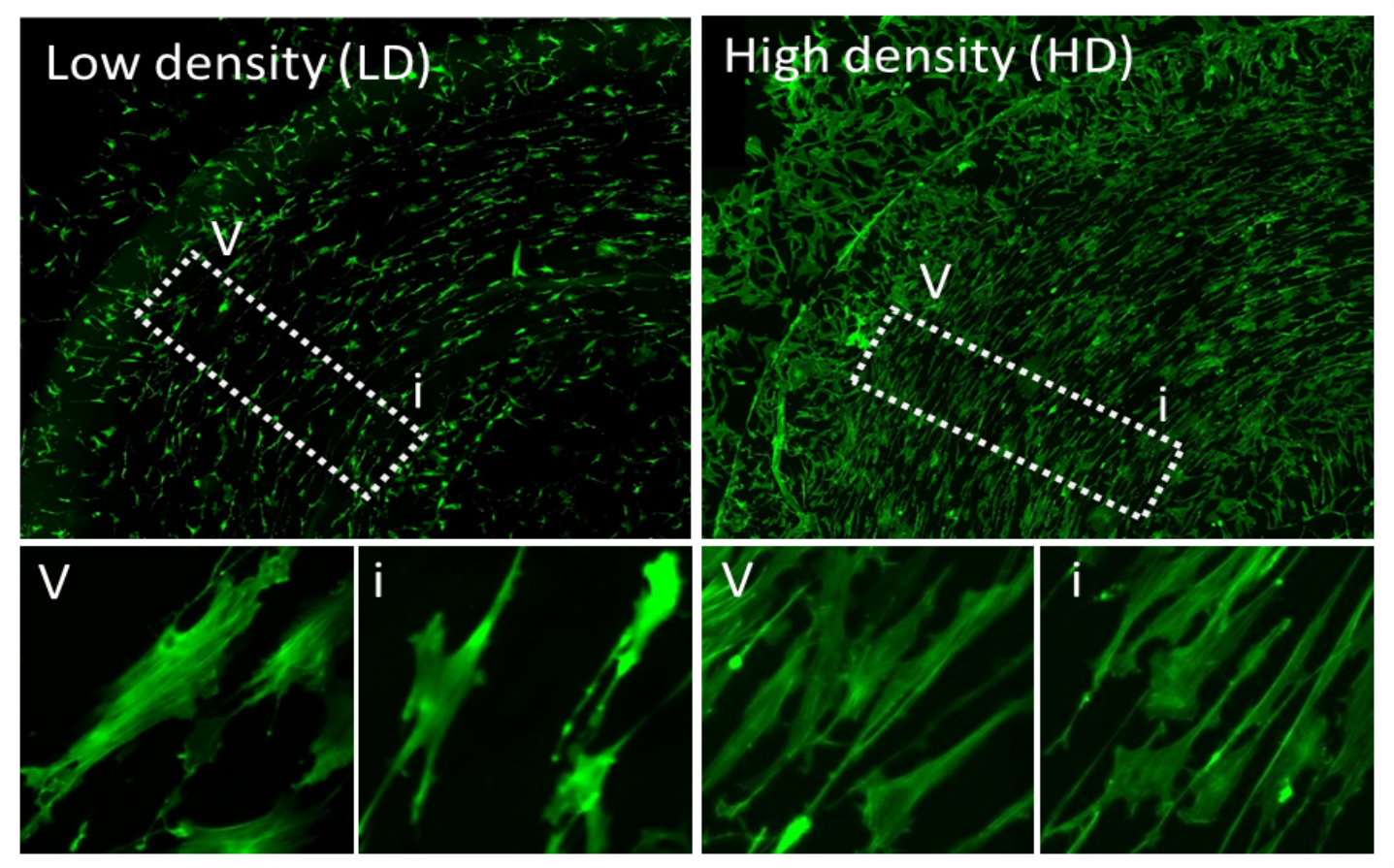


**Supplementary Figure 2.** The differences in collagen wavelength motifs in the concentric area did not affect cell shape, irrespective of the cell seeding density (low density in the left panel; high density in the right panel).
